# Supplementary material for: Berry By-Products in Combination with Antimicrobial Lactic Acid Bacteria Strains for the Sustainable Formulation of Chewing Candies
Source: Foods. 2022 Apr 19;11(9):1177. doi: 10.3390/foods11091177 (PMC9102268; doi:10.3390/foods11091177)
Supplement: Supplementary file 1 [file foods-11-01177-s001.zip › foods-1668145-supplementary.pdf]

Supplementary Materials

Table S1. Antimicrobial activity of lactic acid bacteria against pathogenic and opportunistic strains

Samples	Diameter of inhibition zone, mm									
	Pathogenic and opportunistic strains									
	Pat1	Pat2	Pat3	Pat4	Pat5	Pat6	Pat7	Pat8	Pat9	Pat10
	Lactic acid bacteria									
LUHS135	12.6 ±0.4^{c}	18.3 ±0.4^{c}	15.0 ±0.2^{b}	nd	10.3 ±0.2^{a}	19.1 ±0.3^{b}	20.1 ±0.2^{a}	15.0 ±0.3^{a}	15.1 ±0.2^{a}	48.0 ±0.4^{d}
LUHS244	11.1 ±0.4^{b}	14.5 ±0.2^{a}	14.1 ±0.4^{a}	12.1 ±0.2^{a}	13.2 ±0.4^{b}	20.1 ±0.2^{c}	24.4 ±0.3^{c}	17.0 ±0.2^{b}	16.0 ±0.4^{b}	43.5 ±0.5^{c}
LUHS245	13.3 ±0.2^{d}	16.0 ±0.2^{b}	16.3 ±0.5^{c}	16.1 ±0.3^{c}	20.0 ±0.5^{c}	21.5 ±0.3^{d}	nd	19.5 ±0.4^{d}	16.4 ±0.3^{b}	22.0 ±0.2^{a}
LUHS29	10.1 ±0.3^{a}	14.6 ±0.3^{a}	15.3 ±0.2^{b}	12.7 ±0.3^{b}	nd	10.4 ±0.2^{a}	22.0 ±0.4^{b}	18.1 ±0.4^{c}	15.0 ±0.2^{a}	26.4 ±0.3^{b}

Supercripts $^{a–d}$ - Mean values with different letters between lines are significantly different ($p \leq 0.05$). nd - not detected. LUHS135 - *Lactobacillus plantarum*; LUHS245 - *Lactobacillus uvarum;* LUHS244- *Lactobacillus paracasei*; LUHS29 - *Pediococcus acidilactici.*

Pat1 - *Salmonella enterica*; Pat2 - *Pseudomonas aeruginosa*; Pat3 - Methicillin resistant *Staphylococcus aureus* MRSA M87fox; Pat4 - *Enterococcus faecalis*; Pat5 - *Enterococcus faecium*; Pat6 - *Bacillus cereus*; Pat7 - *Streptococcus mutans*; Pat8 - *Staphylococcus epidermis*; Pat9 - *Staphylococcus haemolyticus*; Pat10 - *Pasteurella multocida*.
